# Influence of tangential displacement on the adhesion strength of a contact between a parabolic profile and an elastic half-space

**DOI:** 10.1098/rsos.161010

**Published:** 2017-08-30

**Authors:** Valentin L. Popov, Iakov A. Lyashenko, Alexander E. Filippov

**Affiliations:** 1 Institut für Mechanik, FG Systemdynamik und Reibungsphysik, Technische Universität Berlin, Sekr. C8-4, Raum M 122, Straße des 17. Juni 135, 10623 Berlin, Germany; 2 National Research Tomsk State University, 634050 Tomsk, Russia; 3 National Research Tomsk Polytechnic University, 634050 Tomsk, Russia; 4 Department of Applied Mathematics and Complex Systems Modelling, Faculty of Electronics and Informational Technologies, Sumy State University, 40007 Sumy, Ukraine; 5 National Academy of Science, Donetsk Institute for Physics and Engineering, 83114 Donetsk, Ukraine

**Keywords:** adhesion, friction, tribology, shear force, numerical simulation, method of dimensionality reduction

## Abstract

The adhesion strength of a contact between a rotationally symmetric indenter and an elastic half-space is analysed analytically and numerically using an extension of the method of dimensionality reduction for superimposed normal/tangential adhesive contacts. In particular, the dependence of the critical adhesion force on the simultaneously applied tangential force is obtained and the relevant dimensionless parameters of the problem are identified. The fracture criterion used coincides with that suggested by Johnson. In this paper, it is used to develop a method that is applicable straightforwardly to adhesive contacts of arbitrary bodies of revolution with compact contact area.

## 1. Introduction

Johnson, Kendall and Roberts developed in 1971 their classical theory of normal adhesive contact between two parabolic, isotropic elastic bodies (JKR theory) [1] using the similarity between the boundary of an adhesive contact and the tip of a mode I crack (opening mode). They applied the same idea of the energy balance that Griffith used in his classical theory of cracks [2]. In the subsequent years, the theory of adhesive contacts developed rapidly [3], mostly using various concepts developed in fracture mechanics.

In the JKR theory—just as in the theory of Griffith—the equilibrium configuration of an adhesive contact is determined by minimizing the total energy of the system including the energy of elastic deformation of contacting bodies, the interface energy and the work of external forces [4]. As this energy does not depend on the tangential displacement, the adhesive contact formally has no ‘tangential strength’. This apparently contradicts experimental observations. The contradiction is due to the microscopically heterogeneous structure of any interface (at the atomic scale, if not earlier), which provides finite contact strength in the tangential direction.

From the microscopic point of view, one can interpret the fracture criterion of Griffith as the requirement that the stress at a fixed distance (of atomic order) from the actual ‘crack tip’ reaches some critical value (stress criterion). This condition leads to the macroscopic dependence of the critical stress on crack length, which is identical to relations obtained from the macroscopic energy balance [5]. Alternatively, one could require that the relative displacement of the faces of the crack achieve some critical value (deformation criterion). The stress and deformation criteria are equivalent for purely elastic bodies, but can lead to different fracture conditions in elastomers [5]. In this paper, we will only deal with purely elastic bodies, so we can apply either the stress or the deformation criterion without loss of generality. Johnson studied the problem of adhesive contact under superimposed normal and tangential loading [6] and concluded that, ‘when tangential forces are applied to an adhesive contact the consequences are not at all well understood’. This situation has not changed much until now.

In his paper of 1997, Johnson approaches the problem of adhesive contact under superimposed normal and tangential loading by considering the complete energy release rate at the boundary of an adhesive contact [6]

1.1 $$Q=\frac{1}{2E^{*}}\left[K_{I}^{2}+K_{\mathrm{II}}^{2}+\frac{1}{1-\nu }K_{\mathrm{III}}^{2}\right],$$

where *K*_I_, *K*_II_ and *K*_III_ are the stress concentration factors defined as

1.2 $$K_{I,\mathrm{II},\mathrm{III}}=\frac{F_{I,\mathrm{II},\mathrm{III}}}{2a\sqrt{\pi a}},$$

with *a* being the contact radius and *F*_I*,*II*,*III_ the components of the applied force in the normal (I) and tangential directions (II, radial direction; III, tangential to the boundary line). The problem of a circular contact remains axially symmetric only if the Poisson number is zero, *ν* = 0. In this case, the stress concentration factors for the modes II and III are equal along the entire boundary line. In the case of arbitrary Poisson number, Johnson suggests to evaluate the average values of *K*_II_ and *K*_III_ around the periphery of the contact area, simplifying (1.1) to

1.3 $$Q=\frac{1}{2E^{*}}\left[K_{I}^{2}+\frac{2-\nu }{2-2\nu }K_{\mathrm{II}}^{2}\right].$$

In terms of energy release rates, the condition of fracture can be formulated by equating the energy release rate to some critical value related to the work of adhesion Δ*γ*. We would like to stress that this approach is by no means self-evident. Physically, it means that elastic energy components due to normal and tangential loading contribute in equal manner to the destruction of interfacial bonds. This may be true in some cases. For example, if some polymer molecules that have to be broken by sufficiently large elongation mediate the adhesion of surfaces, then both normal and tangential deformations have to be considered when applying the ‘fracture criterion’. The same may be valid in a contact of atomically smooth surfaces with equal characteristic range of atomic interaction in normal and tangential directions. In this case, the tangential displacement of atoms at the interface will bring them into a higher energetic position compared with ‘unstressed’ atoms. Subsequently, a smaller amount of work will have to be performed by normal forces to complete the detachment. In this case, too, one can at least qualitatively assume that both normal and tangential parts of elastic energy give approximately the same contribution to the overall detachment energy. In other situations, however, this criterion can fail completely. Thus, if the characteristic range of atomic interactions in the in-plane direction is much smaller than in the normal direction, then the work of adhesion will be practically independent of the tangential loading and the criterion (1.3) will not be valid. One can also imagine a physical model, in which there is some ‘microscopic friction’ between surfaces that are pressed to each other by relatively long-range van der Waals forces. In this case, the work of detachment will depend on the exact ‘direction of detachment’, as was suggested in [7]. Thus, the correct condition for the equilibrium of an adhesive contact under tangential loading cannot be determined from purely theoretical considerations, as it may depend on the specific physics of the interface.

Another important question in considering adhesion is what happens *after* the detachment takes place at some position on the boundary of adhesive contact. If the detachment occurs due to *combined* action of normal and tangential loading, then it may well be the case that the adhesion bonds will be restored after the medium has relaxed the tangential part of elastic energy. This rebinding can actually take place, if it is not prevented by other factors. The simplest such factor may be a rapid change of the surface (e.g. due to oxidation). Another reason may be irreversible changes of surface topography during detachment (so that the surfaces become incongruent and cannot restore the initial configuration). Finally, the actual work of detachment may be much larger than the pure surface energy. In this case, the main part of elastic energy will disappear irreversibly and the relatively weak interfacial interactions will not be able to restore the integrity of the interface again. In all these cases, we would have an *irreversible adhesive contact*.

In this paper, we consider exactly this case of irreversible adhesion. One can interpret this case as a fracture problem of initially glued contact.

In the following, we do not consider the physics of the interface in detail, but just make assumptions similar to those of Johnson, and use the method of dimensionality reduction (MDR) for analysis of critical detachment conditions in analogy to the MDR formulation for the normal adhesive contact [8]. In a series of papers, Popov and co-authors have shown that contact problems of axially symmetric three-dimensional bodies (under the additional assumption of compact contact area) can be equivalently represented by contacts with one-dimensional series of independent springs [5]. It is important to note that the results for axially symmetric contacts obtained with MDR are *exact*, and not an approximation, as is often believed. The MDR was first proposed 2007 for non-adhesive contacts [9], in which case it simply coincides with the solution of Galin & Sneddon [10,11]. In his dissertation of 2011, Heß derived the MDR formulation for adhesive contacts of arbitrary axis-symmetric bodies [12]. A short review of the MDR for contacts of bodies of revolution can be found in [8].

Let us briefly mention previous approaches to the problem of adhesion under superimposed normal and tangential load. In [13], the authors used the discrete element method for modelling contacts between cohesive, frictional particles with normal and tangential loading taking into account adhesion forces between the particles. In [14], a model of tangential adhesion contact was proposed, which, however, requires the assumption that the effects of normal and tangential force can be considered independently. In this investigation, the authors showed that in the tangential contact problem the influence of adhesion can be approximately described in terms of equivalent load. In a series of articles by Guduru and co-authors [15–18] the work of adhesion was considered as a function of ‘mode-mixing’, which means that the work of adhesion depends on the direction of detachment [7,19]. In [15], the theory of Guduru *et al.* was verified experimentally. Tangential adhesion effects were investigated numerically within the framework of coupled Eulerian–Lagrangian method in [20]. In [21], adhesion-induced plastic deformation due to tangential loading was considered. Tangential adhesion effects have been investigated in the context of biological systems [22,23] and physics of particle interactions [13,24,25].

This paper is organized as follows. In §2, we recapitulate briefly the MDR approach for normal adhesive contacts and extend it for the case of superimposed normal and tangential loading under load-controlled and displacement-controlled conditions. The model is then studied numerically and analytically and the dependence of the adhesive force on the tangential force is established in proper dimensionless variables. In §3, we describe the numerical procedure in detail and compare the numerical and analytical results. Section 4 concludes the paper.

## 2. Method of dimensionality reduction formulation for adhesive contact and analytical solution

### 2.1. Method of dimensionality reduction for normal adhesive contacts

We start our consideration with a short introduction to the MDR. Let us consider a contact between an elastic continuum and a rigid, axially symmetric indenter having the shape *f*(*r*), where *r* is the polar radius in the contact plane. If the penetration depth *d* is known as a function of the radius *a* of the contact:

2.1 d=g(a),

then the normal force *F_N_* can be represented as a function of penetration depth by the trivial equation

2.2 $$\begin{array}{rl} F_{N} & =\int_{0}^{F_{N}}\text{d}{\tilde{F}}_{N}=\int_{0}^{a}\frac{\text{d}{\tilde{F}}_{N}}{\text{d}\tilde{d}}\frac{\text{d}\tilde{d}}{\text{d}\tilde{a}}\text{d}\tilde{a}=\int_{0}^{a}k(\tilde{a})\frac{\text{d}g(\tilde{a})}{\text{d}\tilde{a}}\text{d}\tilde{a}\\ & =\int_{0}^{a}\frac{\text{d}k(\tilde{a})}{\text{d}\tilde{a}}(d-g(\tilde{a}))\text{d}\tilde{a}, \end{array}\}$$

where *k*(*ã*) is the stiffness of a cylindrical punch with radius *ã*. Equations (2.1) and (2.2) can be interpreted as the result of the indentation of a modified profile *g*(*ã*) into an elastic foundation with independent springs with spacing d*ã* and stiffness $(1\text{/}2)(\text{d}k(\tilde{a})\text{/d}\tilde{a})\text{d}\tilde{a}$. This interpretation is the basis of the MDR for both homogeneous [8] and non-homogeneous media [26]. In accordance with equations (2.1) and (2.2), the use of MDR is possible under two conditions: (i) the contact stiffness *k*(*ã*) of a cylindrical stamp with a radius *ã* must be known and (ii) the rule of determining the modified profile *g*(*ã*) is known. Depending on the circumstances, these two steps may be performed analytically, numerically or experimentally.

**Figure 1.**
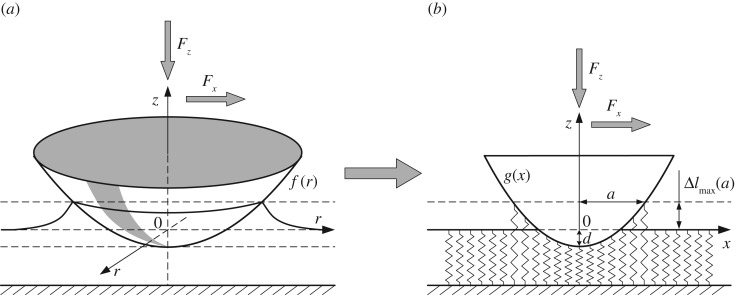
MDR transformation of (*a*) the original three-dimensional profile *f*(*r*) into (*b*) a one-dimensional image *g*(*x*) and replacement of the elastic half-space by an elastic foundation. In the presence of normal and tangential force and adhesion, the springs of the elastic foundation will be displaced both in the normal and tangential directions. In this figure, only vertical displacements are shown.

For homogeneous media, the rule for finding the modified profile *g*(*ã*) is known explicitly. In the following, we will denote the argument of this function by *x* as is usually done in the MDR. However, in this context *x* does not denote any spacial coordinate but the internal variable of the MDR. The initial three-dimensional profile *f*(*r*) (shown in figure 1*a*) is first replaced by the one-dimensional profile *g*(*x*) by means of the transformation [8]:

2.3 $$g(x)=|x|\int_{0}^{\left|x\right|}\frac{f^{\prime }(r)}{\sqrt{x^{2}-r^{2}}}\text{d}r.$$

If needed, the original surface *z* = *f*(*r*) can be always restored from its MDR-transformed one-dimensional profile by

2.4 $$f(r)=\frac{2}{\pi }\int_{0}^{r}\frac{g(x)}{\sqrt{r^{2}-x^{2}}}\text{d}x.$$

In this paper, we will limit ourselves to parabolic profiles of the form *f*(*r*) = *r*^2^/(2*R*). However, generalization to arbitrary rotationally symmetric profiles is straightforward. In the case of the parabolic profile, transformation (2.3) leads again to a parabolic profile *g*(*x*) with a changed coefficient:

2.5 $$f(r)=\frac{r^{2}}{2R}\;\Rightarrow \;g(x)=\frac{x^{2}}{R}.$$

In the second step [8], the elastic half-space must be replaced by an elastic foundation, as shown in figure 1, consisting of independent springs having normal and tangential stiffness

2.6 $$k_{z}=E^{*}\Delta x\;\text{and}\;k_{x}=G^{*}\Delta x,$$

where Δ*x* is the spacing of the springs and the effective moduli E* and G* are defined as

2.7 $$E^{*}=\frac{E}{1-\nu^{2}}=\frac{2G}{1-\nu },\;G^{*}=\frac{4G}{2-\nu },$$

so that

2.8 $$G^{*}=E^{*}\frac{2-2\nu }{2-\nu }.$$

Note that in the case of *ν* = 0 both effective moduli coincide: E* = G*. For definiteness and simplicity, all numerical simulations and analytical calculations below are performed under this assumption.

Before proceeding to tangential contacts, we will first recapitulate the application of the MDR to normal adhesive contacts. If the MDR-transformed profile *g*(*x*) is indented into the elastic foundation by an indentation depth *d*, then the displacement of individual springs inside the contact will be determined by the equation

2.9 $$u_{z}(x)=d-g(x)=d-\frac{x^{2}}{R}.$$

The size of the adhesive contact at a given indentation depth can be easily found from the principle of virtual work: the springs at the boundary of contact are stretched by $\Delta l=-u_{z}(a)$. The energy released through detachment of two boundary springs is equal to $E^{*}\Delta l^{2}\Delta x$. Through detachment, the free surface energy 2πaΔxΔγ is created (this energy can only be defined in the original, three-dimensional system). According to the principle of virtual work, the system will be in equilibrium if these two energies are equal:

2.10 $$E^{*}\Delta l^{2}\Delta x=2\pi a\Delta x\Delta \gamma .$$

It follows that the condition of equilibrium of boundary springs can be written as

2.11 $$\Delta l=\Delta l_{\max}(a)=\sqrt{\frac{2\pi a\Delta \gamma }{E^{*}}}.$$

This condition is known as the *rule of Heß* [12]. Combining (2.9) and (2.11), we get

2.12 $$u_{z}(a)=d-\frac{a^{2}}{R}=-\Delta l_{\max}(a)=-\sqrt{\frac{2\pi a\Delta \gamma }{E^{*}}}$$

or

2.13 $$d=\frac{a^{2}}{R}-\sqrt{\frac{2\pi a\Delta \gamma }{E^{*}}}.$$

The normal force can be calculated as the sum of all spring forces:

2.14 $$F_{z}(a)=E^{*}\int_{-a}^{a}u_{z}(x)\text{d}x=2E^{*}\int_{0}^{a}\left(d-\frac{x^{2}}{R}\right)\text{d}x=\frac{4E^{*}a^{3}}{3R}-\sqrt{8\pi a^{3}E^{*}\Delta \gamma }.$$

Later we will consider a more general situation, where the indenter is also displaced in the tangential direction by $u_{x}^{\left(0\right)}$. It is convenient to present both analytical and numerical results in terms of dimensionless variables:

2.15 $$\tilde{a}=\frac{a}{a_{0}},\;{\tilde{F}}_{z}=\frac{F_{z}}{F_{0}},\;\tilde{d}=\frac{d}{d_{0}},\;{\tilde{u}}_{x}^{\left(0\right)}=\frac{u_{x}^{\left(0\right)}}{d_{0}},\;{\tilde{u}}_{z}=\frac{u_{z}}{d_{0}},$$

where *F*_0_, *a*_0_ and *d*_0_ are the critical values of the force, the contact radius and the absolute indentation depth at the moment of detachment of the parabolic profile from the elastic half-space under force-controlled conditions [4]:

2.16 $$F_{0}=\frac{3}{2}\pi R\Delta \gamma ,\;a_{0}={\left(\frac{9\pi R^{2}\Delta \gamma }{8E^{*}}\right)}^{1/3},\;d_{0}={\left(\frac{3\pi^{2}R\Delta \gamma^{2}}{64{E^{*}}^{2}}\right)}^{1/3}.$$

In dimensionless variables, equations (2.13) and (2.14) take the form

2.17 $$\tilde{d}=3{\tilde{a}}^{2}-4{\tilde{a}}^{1/2}$$

and

2.18 $$\tilde{F}={\tilde{a}}^{3}-2{\tilde{a}}^{3/2}.$$

**Figure 2.**
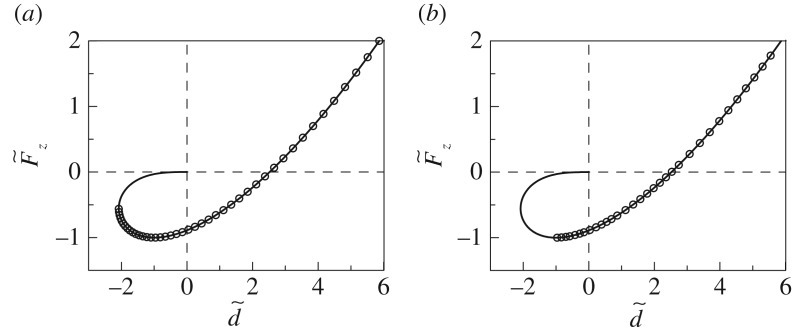
Dependence of the normal force on the indentation depth for the normal contact with adhesion. Solid lines show the analytical solution defined by equations (2.17) and (2.18). Circles represent results of numerical experiments for displacement-controlled (*a*) and load-controlled (*b*) conditions as described in §3.

These results of course coincide with the classical solution of Johnson *et al.* [1]. The dependence of the dimensionless normal force on the dimensionless approach (indentation depth) implicitly defined by equations (2.17) and (2.18) is shown in figure 2 and will be used for testing numerical procedures described in §3.

We now analyse the condition of instability of the contact, i.e. the conditions under which the possibility of the adhesive contact to sustain equilibrium is lost. We will consider both displacement-controlled and load-controlled contacts. Under displacement-controlled conditions, the macroscopic displacement of the indenter is imposed by a very stiff external system. Physically, this means that during the movement of the system towards the equilibrium state, the displacement is kept exactly constant. Load-controlled conditions are physically realized by applying the given force through a very soft spring. Thus, in conditions of load control, the force during the relaxation to the equilibrium remains fixed. The method described in this section has been used successfully for modelling the influence of adhesion on impact between elastic particles under displacement-controlled conditions [25].

### 2.2. Superimposed normal and tangential loading

Let us now assume that the loading of the profile consists of superimposed normal force and tangential displacement $u_{x}^{\left(0\right)}$. The energy released by detachment of two boundary springs will now be equal to $E^{*}u_{z}{\left(a\right)}^{2}\Delta x+G^{*}u_{x}^{(0)2}\Delta x$. Equating this to the work of adhesion 2πaΔxΔγ, we arrive at the following equilibrium condition:

2.19 $$E^{*}u_{z}{\left(a\right)}^{2}+G^{*}u_{x}^{(0)2}=2\pi a\Delta \gamma .$$

This rule is exactly equivalent to the rule obtained by Johnson on the basis of the energy release rate (1.3). From (2.19), for the elongation of the boundary springs we get

2.20 $$|u_{z}(a)|=\sqrt{\frac{2\pi a\Delta \gamma }{E^{*}}-\frac{G^{*}}{E^{*}}u_{x}^{(0)2}}.$$

Using (2.9), we can write the relationship between the indentation depth and contact radius in the form

2.21 $$d=\frac{a^{2}}{R}-\sqrt{\frac{2\pi a\Delta \gamma }{E^{*}}-\frac{G^{*}}{E^{*}}u_{x}^{(0)2}}.$$

The normal and tangential forces are functions of contact radius *a*:

2.22 $$F_{z}=2E^{*}\left(ad-\frac{a^{3}}{3R}\right)$$

and

2.23 $$F_{x}=2G^{*}a\cdot u_{x}^{\left(0\right)}.$$

In dimensionless variables (2.15), equations (2.21)–(2.23) can be written as

2.24 $$\begin{array}{rl} \tilde{d} & =3{\tilde{a}}^{2}-\sqrt{16\tilde{a}-\frac{G^{*}}{E^{*}}{\tilde{u}}_{x}^{(0)2}}, \end{array}$$

2.25 $$\begin{array}{rl} {\tilde{F}}_{z} & =\frac{\tilde{a}}{2}(\tilde{d}-{\tilde{a}}^{2}) \end{array}$$

2.26 $$\begin{array}{rl} \;\text{and}\;{\tilde{F}}_{x} & =\frac{G^{*}}{2E^{*}}\tilde{a}\cdot {\tilde{u}}_{x}^{\left(0\right)}. \end{array}$$

These equations determine the normal force–indentation relation in the presence of tangential displacement. Note that substitution of (2.24) into (2.25) at ${\tilde{u}}_{x}^{\left(0\right)}=0$ reduces to the result (2.18) for the normal contact.

Let us stress that equation (2.19) assumes that the work of adhesion does not depend on the direction in which the surfaces are detached from each other. This is a physical assumption that may be incorrect in some systems [7,19]. At this point, further investigation of the process of detachment would have to be carried out. In the following, we remain in the framework of the ‘Johnson paradigm’, and use the detachment condition (2.19) and equations based on it. We will treat both the displacement-controlled and load-controlled cases in the normal direction, but will confine ourselves to displacement control in the horizontal direction.

#### 2.2.1. Adhesion force under load-controlled conditions

Under load-controlled conditions, the instability occurs at the contact radius at which the normal force is minimized. Thus, the condition of instability can be written as d*F_z_*/d*a* *=* 0. Differentiating equation (2.22) with respect to *a* and using equation (2.21) we arrive at the condition

2.27 $$\frac{2a_{c,fl}^{2}}{R}\sqrt{\frac{2\pi \Delta \gamma a_{c,fl}}{E^{*}}-\frac{G^{*}}{E^{*}}u_{x}^{(0)2}}-\frac{3\pi \Delta \gamma a_{c,fl}}{E^{*}}+\frac{G^{*}}{E^{*}}u_{x}^{(0)2}=0,$$

or, in dimensionless variables (2.15)

2.28 $${\tilde{a}}_{c,fl}^{2}\sqrt{{\tilde{a}}_{c,fl}-\frac{G^{*}}{16E^{*}}{\tilde{u}}_{x}^{(0)2}}-{\tilde{a}}_{c,fl}+\frac{G^{*}}{24E^{*}}{\tilde{u}}_{x}^{(0)2}=0.$$

**Figure 3.**
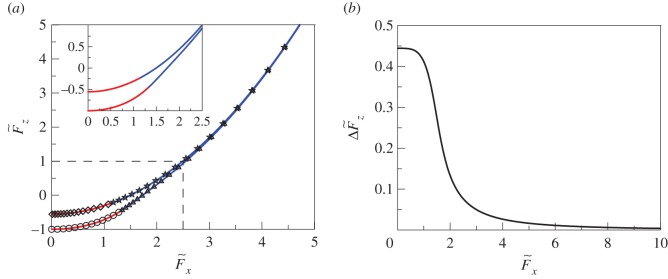
(*a*) The dependence of normalized critical normal force ${\tilde{F}}_{z}$ on normalized critical tangential force ${\tilde{F}}_{x}$, for the case E* = G*. Analytical results are represented by solid lines and the results of numerical simulation by open circles, diamonds, stars and triangles. The upper line (diamonds and stars) corresponds to displacement control in both directions. The lower line (open circles and triangles) corresponds to load control in the vertical direction and displacement control in the tangential direction. Diamonds and open circles (red lines) correspond to detachment at a negative indentation depth *d*. Stars and triangles correspond to detachment at a positive indentation depth; (*b*) the difference between the normal forces shown in (*a*) as a function of normalized tangential force ${\tilde{F}}_{x}$.

This equation determines the dependence of the critical radius ${\tilde{a}}_{c,fl}$ on the tangential displacement ${\tilde{u}}_{x}^{\left(0\right)}$. The dependence of the adhesion force on the tangential force can be determined by substituting ${\tilde{a}}_{c,fl}$ into equations (2.24)–(2.26). This dependence is shown in figure 3*a* (lower solid line). For ${\tilde{F}}_{x}=0$ (or ${\tilde{u}}_{x}^{\left(0\right)}=0$), equations (2.24), (2.25) and (2.28) provide the critical force ${\tilde{F}}_{z}(0)=-1$.

Let us note that the dependence shown in figure 3*a* determines the critical tangential force for both positive and negative normal forces. In the case of the negative normal force, this critical value really corresponds to loss of ‘adhesive’ contact, as destruction of the contact will lead to movement of the indenter away from the substrate. In the case of positive normal forces, contact will not be lost at the critical value, only the continuity of the contact will be lost, which can be understood as propagation of a mode II (tangential) crack. The critical condition (2.19) does not differentiate between these two cases, so the resulting dependences of *F_z_* on *F_x_* are equally valid for positive and negative *F_z_*, although the physical interpretation is different.

#### 2.2.2. Adhesion under displacement-controlled conditions

For the pure normal contact, this mode is shown in figure 2*a*; the detachment occurs at $F_{z}/F_{0}={\tilde{F}}_{z}=-0.5$. The condition of instability is in this case formally given by d(*d*)/d*a* = 0. Thus, in the general case of superimposed normal and tangential loading, differentiating (2.24) gives

2.29 $$a_{c,fg}^{3}-\frac{G^{*}u_{x}^{(0)2}}{2\pi \Delta \gamma }a_{c,fg}^{2}-\frac{2\pi \Delta \gamma R^{2}}{16E^{*}}=0,$$

or, in dimensionless variables (2.15)

2.30 $${\tilde{a}}_{c,fg}^{3}-\frac{G^{*}}{16E^{*}}{\left({\tilde{u}}_{x}^{\left(0\right)}{\tilde{a}}_{c,fg}^{}\right)}^{2}-\frac{1}{9}=0.$$

This equation determines the dependence of the critical radius ${\tilde{a}}_{c,fg}$ on the tangential displacement ${\tilde{u}}_{x}^{\left(0\right)}$. For critical forces at the moment of detachment, we obtain

2.31 $${\tilde{F}}_{x}^{2}=\frac{4G^{*}}{E^{*}}\left({\tilde{a}}^{3}-\frac{1}{9}\right)$$

and

2.32 $${\tilde{F}}_{z}={\tilde{a}}^{3}-\frac{2}{3}.$$

From the two last equations, it follows

2.33 $${\tilde{F}}_{z}=\frac{E^{*}}{4G^{*}}{\tilde{F}}_{x}^{2}-\frac{5}{9}.$$

For the case E* = G*, the relationship between ${\tilde{F}}_{z}$ and ${\tilde{F}}_{x}^{}$ under displacement control simplifies to

2.34 $${\tilde{F}}_{z}=\frac{1}{4}{\tilde{F}}_{x}^{2}-\frac{5}{9}.$$

This dependence is shown in figure 3*a* (upper solid line). Additionally, in figure 3*b* the differences between the normal forces shown in figure 3*a*, $\Delta {\tilde{F}}_{z}$, are shown as a function of normalized tangential force ${\tilde{F}}_{x}$. At zero tangential force ${\tilde{F}}_{x}=0$, this difference is equal to $\Delta {\tilde{F}}_{z}(0)={\tilde{F}}_{z}^{fg}(0)-{\tilde{F}}_{z}^{fl}(0)=-5\text{/}9-(-1)=4\text{/}9$. With increasing ${\tilde{F}}_{x}$ the value $\Delta {\tilde{F}}_{z}$ monotonically decreases. Note that the lower dependence in figure 3*a* (under load-controlled conditions in the normal direction and displacement control in the tangential direction) is well approximated by equation (2.34) when value $\Delta {\tilde{F}}_{z}$ shown in figure 3*b* is close to zero. At ${\tilde{F}}_{x}\gg 1$ both dependences shown in figure 3*a* coincide and are described by equation (2.34).

## 3. Numerical procedure and comparison with analytical results

In the following, we reproduce the above results numerically and use the developed numerical procedure to extend them to a more complicated detachment condition. Let us first briefly describe the numerical procedure for the case of normal adhesive contact. In the first step, the modified parabolic profile *g*(*x*) (2.5) is indented by *d* into the elastic foundation shown in figure 1*b*. After this, the position of the critical boundary springs (and thus the contact radius) is calculated using the condition (2.11). Given the indentation depth and the contact radius, the normal and tangential forces can be obtained by summing the forces of all springs in contact:

3.1 $$F_{z}=E^{*}\Delta x\sum_{\mathrm{cont}}u_{z}(x_{i})$$

and

3.2 $$F_{x}=G^{*}\Delta x\sum_{\mathrm{cont}}u_{x}(x_{i}),$$

where *u_z_*(*x_i_*) and *u_x_*(*x_i_*) are, respectively, the normal and tangential displacements of individual springs at the coordinate *x_i_*. In a purely normal contact *u_x_*(*x_i_*) = 0, and *F_x_* = 0.

When analysing the adhesive contact under displacement-controlled conditions, we displace the rigid indenter in the vertical direction step by step with a chosen discretization Δ*z*. The new configuration of contact after each step is calculated using the rule (2.11) for the springs at the boundary of the contact. If it happens that more than two springs are detached, we return to the previous step and proceed further with a discretization step Δ*z*/2 and, if necessary, decrease it further until only one spring is lost on each side. This procedure is continued up to the point of the instability.

For load-controlled conditions, the controlling parameter is the normal force *F*_up_. In each step, *F*_up_ is increased by an increment Δ*F*, and the new equilibrium configuration of the contact is found. Similar to the procedure for displacement control, the increment of the force is decreased if more than two springs are detached in one step. The results of numerical calculations with displacement-controlled and load-controlled conditions for normal motion are presented together with analytical results in figure 2. Since the numerical results coincide with the analytical ones, this provides some validation for the above numerical procedure.

The procedure in the case of combined normal and tangential loading is basically the same as described above. The only difference is the use of a modified detachment condition

3.3 $$\Delta l=\sqrt{u_{x}^{2}+u_{z}^{2}}=\Delta l_{\max}.$$

In figure 3*a*, the results of numerical calculations are shown along with analytical results presented in the previous section.

## 4. Conclusion

We performed analytical and numerical analysis of the adhesive contact between two elastic bodies with an axially symmetric gap profile under superimposed normal and tangential loading. The study was performed under the simplest assumption that the surface energy does not depend on the direction of detachment. However, the developed analytical method can be generalized straightforwardly for more complicated adhesive interactions, as for example suggested in [23]. Under the above assumptions, the application of tangential force leads to a decrease of the normal adhesive force. We considered different combinations of controlled load and controlled displacement in both normal and tangential directions and derived for each case solutions in the appropriate dimensionless variables. In the case of more general adhesive interaction than assumed in this paper, it would further be interesting to take into account the partial slip and frictional forces in the contact plane (as has been done for the special case of the Dugdale adhesive potential in [27]).

## Data accessibility

No supporting data needed. All necessary algorithms are fully described in article.

## References

[1] Johnson KL, Kendall K, Roberts AD 1971 Surface energy and the contact of elastic solids. Proc. R. Soc. Lond. A 324, 301–313. (doi:10.1098/rspa.1971.0141)

[2] Griffith AA 1921 The phenomena of rupture and flow in solids. Phil. Trans. R. Soc. Lond. A 221, 163–198. (doi:10.1098/rsta.1921.0006)

[3] Maugis D 2000 Contact, adhesion, and rupture of elastic solids. Berlin, Germany: Springer.

[4] Popov VL 2017 Contact mechanics and friction. Physical principles and applications. 2nd edn Berlin, Germany: Springer.

[5] Popov VL, Heß M 2015 Method of dimensionality reduction in contact mechanics and friction. Berlin, Germany: Springer.

[6] Johnson KL 1997 Adhesion and friction between a smooth elastic spherical asperity and a plane surface. Proc. R. Soc. Lond. A 453, 163–179. (doi:10.1098/rspa.1997.0010)

[7] Hutchinson JW, Suo Z 1991 Mixed mode cracking in layered materials. Adv. Appl. Mech. 29, 63–191. (doi:10.1016/S0065-2156(08)70164-9)

[8] Popov VL, Hess M 2014 Method of dimensionality reduction in contact mechanics and friction: a user's handbook. I. Axially-symmetric contacts. Facta Universitatis, series Mech. Eng. 12, 1–14. (doi:10.2298/FUACE1401001S)

[9] Popov VL, Psakhie SG 2007 Numerical simulation methods in tribology. Tribol. Int. 40, 916–923. (doi:10.1016/j.triboint.2006.02.020)

[10] Galin LA 1961 Contact problems in the theory of elasticity. Raleigh, NC: North Carolina State College.

[11] Sneddon IN 1965 The relation between load and penetration in the axisymmetric Boussinesq problem for a punch of arbitrary profile. Int. J. Eng. Sci. 3, 47–57. (doi:10.1016/0020-7225(65)90019-4)

[12] Heß M 2011 Über die Abbildung ausgewählter dreidimensionaler Kontakte auf Systeme mit niedrigerer räumlicher Dimension. Göttingen, Germany: Cuvillier.

[13] Luding S 2008 Cohesive, frictional powders: contact models for tension. Granular Matter 10, 235–246. (doi:10.1007/s10035-008-0099-x)

[14] Qunyang L, Shouwen Y 2004 A model for computational investigation of elasto-plastic normal and tangential contact considering adhesion effect. Acta Mech. Sin. 20, 165–171. (doi:10.1007/BF02484261)

[15] Waters JF, Guduru PR 2010 Mode-mixity-dependent adhesive contact of a sphere on a plane surface. Proc. R. Soc. A 466, 1303–1325. (doi:10.1098/rspa.2009.0461)

[16] Waters JF, Guduru PR 2011 A mechanism for enhanced static sliding resistance owing to surface waviness. Proc. R. Soc. A 467, 2209–2223. (doi:10.1098/rspa.2010.0617)

[17] Waters JF, Kalow J, Gao H, Guduru PR 2012 Axisymmetric adhesive contact under equibiaxial stretching. J. Adhes. 88, 134–144. (doi:10.1080/00218464.2012.648061)

[18] Waters JF, Gao HJ, Guduru PR 2011 On adhesion enhancement due to concave surface geometries. J. Adhes. 87, 194–213. (doi:10.1080/00218464.2011.557325)

[19] Kim K-S, McMeeking RM, Johnson KL 1998 Adhesion, slip, cohesive zones and energy fluxes for elastic spheres in contact. J. Mech. Phys. Solids 46, 243–266. (doi:10.1016/S0022-5096(97)00070-7)

[20] Lorentz B, Albers A 2013 A numerical model for mixed lubrication taking into account surface topography, tangential adhesion effects and plastic deformations. Tribol. Int. 59, 259–266. (doi:10.1016/j.triboint.2012.08.023)

[21] Maloney JM, Walton EB, Bruce CM, Van Vliet KJ 2008 Influence of finite thickness and stiffness on cellular adhesion-induced deformation of compliant substrata. Phys. Rev. E 78, 041923 (doi:10.1103/PhysRevE.78.041923) 10.1103/PhysRevE.78.041923 18999471

[22] Terekhov AV, Hayward V 2011 Minimal adhesion surface area in tangentially loaded digital contacts. J. Biomech. 44, 2508–2510. (doi:10.1016/j.jbiomech.2011.07.007) 21774936 10.1016/j.jbiomech.2011.07.007

[23] Lyashenko IA 2016 Influence of tangential displacement on critical normal force of adhesive contact breakage in biological systems. Facta Universitatis, series: Mech. Eng. 14, 313–320. (doi:10.22190/FUME1603313L)

[24] Parent JR, Adams GG 2015 Adhesion-induced tangential driving force acting on a spherical particle lying on a sinusoidal surface. J. Adhes. 92, 273–281. (doi:10.1080/00218464.2015.1026333)

[25] Lyashenko IA, Willert E, Popov VL 2016 Adhesive impact of an elastic sphere with an elastic half space: numerical analysis based on the method of dimensionality reduction. Mech. Mater. 92, 155–163. (doi:10.1016/j.mechmat.2015.09.009) 10.1038/srep08479 PMC4329545 25684339

[26] Hess M, Popov VL 2016 Method of dimensionality reduction in contact mechanics and friction: a user's handbook. II. Power-law graded materials. Facta Universitatis, series: Mech. Eng. 14, 251–268. (doi:10.22190/FUME1603251H)

[27] Popov VL, Dimaki AV In press Friction in an adhesive tangential contact in the Coulomb-Dugdale approximation. J. Adhes. (doi:10.1080/00218464.2016.1214912)

